# Automated Risk Control in Medical Imaging Equipment Management Using Cloud Application

**DOI:** 10.1155/2018/7125258

**Published:** 2018-05-02

**Authors:** Sally M. Ghanem, Manal Abdel Wahed, Neven Saleh

**Affiliations:** ^1^Systems and Biomedical Engineering Department, Faculty of Engineering, Cairo University, Giza, Egypt; ^2^Systems and Biomedical Engineering Department, Higher Institute of Engineering in El-Shorouk city, Cairo, Egypt

## Abstract

Medical imaging equipment (MIE) is the baseline of providing patient diagnosis in healthcare facilities. However, that type of equipment poses high risk for patients, operators, and environment in terms of technology and application. Considering risk management in MIE management is rarely covered in literature. The study proposes a methodology that controls risks associated with MIE management. The methodology is based on proposing a set of key performance indicators (KPIs) that lead to identify a set of undesired events (UDEs), and through a risk matrix, a risk level is evaluated. By using cloud computing software, risks could be controlled to be manageable. The methodology was verified by using a data set of 204 pieces of MIE along 104 hospitals, which belong to Egyptian Ministry of Health. Results point to appropriateness of proposed KPIs and UDEs in risk evaluation and control. Thus, the study reveals that optimizing risks taking into account the costs has an impact on risk control of MIE management.

## 1. Introduction

Risk management becomes an essential concept to ensure safety, reliability, competence, and compliance with standards in healthcare facilities. As medical equipment poses risks to patients, users, and environment, The Joint Commission (TJC) integrates elements related to risk management into its standards. In 2004, TJC issued risk management standards that require hospitals to adhere that “*The organization manages medical equipment risks*” and “*Medical equipment is maintained, tested, and inspected*” [1].

Risk management process starts with identifying potential hazards and then assessing the *likelihood of occurrence* and *severity* of each hazard. In order to evaluate risks associated with each hazard, a risk number (RN) is calculated, and then based on this number, risks are ranked to be controlled [2–4]. In fact, to recognize a hazard or an undesired event (UDE), a set of key performance indicators (KPIs) should be identified to monitor and measure associated risks for any process [5, 6].

In this context, several researches have been conducted to study risk management for medical equipment. For instance, Dumbrique discussed risk management in medical equipment manufacturing process [7]. Another aspect was presented in [3], concerning risk management in software industry. Another approach has been proposed by Tawfik, Ouda, and Abd El Samad to classify risk levels of medical equipment taking into account the operational and environmental conditions [8].

Although medical imaging equipment (MIE) is the front line of the basic diagnosis in healthcare services, no doubt that it is considered as a source of high risk in terms of technology and application. Almost, if not all, studies that have been presented in MIE for risk management regarded the radiation safety, overdose control, and electrical hazards [9–11]. Yet, controlling risks associated with poor management of MIE especially for developing countries is rarely considered in literature. In addition, optimization of the balance between risks and costs is an essential requirement [12].

The goal of this study is to manage and control risks associated with MIE due to poor management and low utilization rate. This is achieved by identifying a worthy KPI set for MIE management that leads to recognize UDEs of the management process. Consequently, risk management process takes place as described before. Due to no specific KPIs for medical equipment management, the challenge of this study is to suggest a set of KPIs and UDEs for risks associated with MIE management.

The rest of the article is organized as follows: the methodologies and procedures to identify KPIs and UDEs for MIE management are described and discussed in Section 2. A case study for validation with results and analysis is adopted and explained in Section 3. Finally, conclusions and future work are presented in Section 4.

## 2. Materials and Methods

In this study, the rules of risk management are conducted for MIE management. Identification of a set of UDEs is adopted, which implies specifying a set of KPIs by setting threshold values of these KPIs. Once UDEs are identified, risk control is carried out by calculating risk number for each UDE that leads to ranking risks and follows appropriate control action. Considering automation approach, a software program is followed for implementation. The interpretation of each step is explained in detail in next sections.

### 2.1. Key Performance Indicators

Key performance indicators are used to measure the performance of individuals and processes. To the best of our knowledge, there are not specific KPIs for medical equipment management. To overcome this problem, the authors decided to take the approach of expert's opinions as well as utilizing the guidelines and the standards of medical equipment maintenance to conclude these KPIs. Such standards are related to International Organization for Standardization (ISO), Food and Drug Administration (FDA), World Health Organization (WHO), and TJC.

According to [5], the KPIs are classified into *three classes*: *purpose* (P) such as standard compliance, *effect* (E) such as plant availability, and *cause* (C) such as work completion. Regarding the preventive maintenance (PM) and corrective maintenance (CM) of medical equipment, the KPIs are proposed as shown in Table 1.

As illustrated in Table 1, CMV stands for corrective maintenance visit and PMV stands for preventive maintenance visit. The KPIs from 1 to 6 are frequency of occurrence of measurable elements. The KPI 7 “mean response time” is the mean elapsed time between raising a complaint and first response visit, whereas the KPI 8 “mean repair time” is the mean elapsed time between the first repair visit and last visit in which the device is turned as before malfunctioning.

KPI 9 and KPI 10 measure “mean time between PM visits per MIE” and “mean time between PMV and CMV per MIE,” respectively. Percentage errors due to data entry are calculated in KPI 11, while missed data percentage is reported for KPI 12. All the KPIs are calculated and reported annually as presented in Table 1.

### 2.2. Undesired Events

For any process, undesired event is an expression about a source of hazard if an activity within a process exceeds its permitted limits. Accordingly, in order to specify UDEs of MIE management, a set of thresholds must be suggested. The KPIs are considered as the platform of UDEs. In this study, 11 UDEs with their thresholds are proposed as shown in Table 2. Thresholds are set for MIE based on experience, personnel judgment, and literature review.

### 2.3. Risk Calculation

Risk management involves evaluating potential hazards *consequences* and their *likelihood of occurrence* to determine risk level. Risk control is a process through which a decision is made based on risk level to mitigate risks to a certain level or to eliminate risks as possible or even tolerate it. Further, a risk matrix is a graphical representation tool used to assess risks visually [4, 14].

In risk matrix, the *probability of occurrence* (*P*) is presented across *the rows*, while the consequence or *severity* (*S*) is presented across *the columns*. Each cell within the matrix is the product number of *P* and *S* that points to a risk number and consequently a risk level. In addition, the matrix is divided into 3 zones: *generally acceptable risk (zone 1), conditionally acceptable risk (zone 2)*, and *generally unacceptable risk (zone 3)* [15]. Each zone is indicated by a separate color to be visually distinguished.

**Figure 1:**
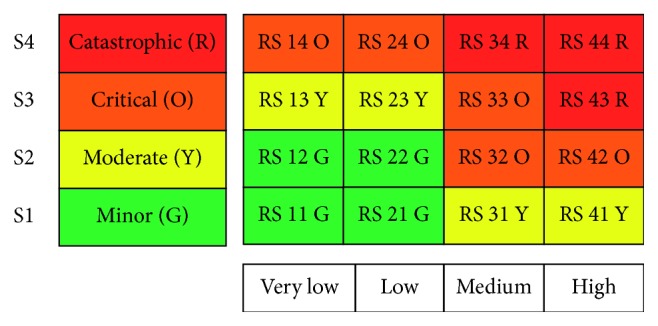
The risk matrix form.

Using these concepts, the risk matrix is established as illustrated in Figure 1. It is a square matrix (4 × 4) in which the probability of occurrence is classified into *very low*, *low*, *medium*, and *high*, whereas the severity is categorized as *minor*, *moderate*, *critical*, and *catastrophic*. The color code is used as follows: green (G) and yellow (Y) cells belong zone 1, orange (O) cells relates to zone 2, and red (R) cells for zone 3.

Because the research concerns MIE management risks, the severity is estimated with respect to MIE *total costs* including purchase price, running cost, and hidden cost. Hence, the severity of each UDE should be calculated in monetary value. Taking into account each UDE, a risk matrix should be established for each one.

#### 2.3.1. Severity

The UDE severity (S) is determined using the following calculations as in (1). In assumption, the annual working days are supposed to be *300 days*, and the expected lifetime is 10 years as well as the hidden cost factor is assumed to be 10.

(1) $$\begin{array}{c} \begin{array}{l} \text{UDE severity}=D\times \text{MDC,}\\ \text{MDC}=\text{MDDC}\times H,\\ \text{MDDC}=\frac{\text{ADDC}}{N},\\ \text{ADDC}=\frac{\text{total cost}}{L\times W}, \end{array} \end{array}$$

where *D* is the downtime (days) in which MIE is out of service, MDC is the machine daily cost, MDDC is the machine daily direct cost, *H* is the hidden cost factor, ADDC is the all daily direct cost, *L* is the expected life time in years, and *W* is the working days per year.

#### 2.3.2. Probability of Occurrence

The probability of occurrence (*P*) is expressed for each UDE as shown in Table 2. Terms identification is described below.

N: total number of MIE

A: total number of MIE with complaints per year

K: total number of MIE with complaints/investigated period

B: total number of MIE with MTB_PMV is <120 days

C: total number of MIE with MTB_PMV is >240 days

M: total number of maintained MIE

T: total repair days per MIE per year

G: total number of MIE with MTB_PMV_CMV is <100 days

E: total errors in data entry

TD: total data items

MD: total missed data items.

### 2.4. Cloud Application

In last few years, cloud applications have been widely used in a way that proves customer trust. It offers a broad range of IT services over the Internet. It covers three types of services: Platform as a Service (*PaaS*), Software as a Service (*SaaS*), and Infrastructure as a Service (*IaaS*). *SaaS* hosts software as a service provided to all users, while IaaS is used for computing control and storage. *PaaS* acts as an integrated solution over the clouds [16, 17].

Unlike IaaS, which provides only infrastructure, *SaaS* is more appropriate for our application because it provides a tailor-made application based on customer requirements. In addition, the service is provided to all users across the network without requiring installation and running of the application on their computers [17, 18].

## 3. Results and Discussion

The methodology verification was carried out employing a data set of medical imaging equipment consisting of 204 pieces of MIE along 104 Egyptian hospitals across 25 governorates managed by Directorate General Radiology (DGR) as an authorized entity of Egyptian Ministry of Health (MOH). The data set belongs to different types of MIE including conventional X-ray, computed tomography (CT), ultrasound, C-Arm, and automated film processors.

The investigated period is 6.25 years through which a data set was collected. The period was starting from January 2008 until March 2014. It is worthy to mention that before January 2011, the equipment was managed manually using paper-based management system. Starting from that date, the MIE set is managed using open-source *SaaS* cloud computing software called open Medical Equipment and Devices Information System (OpenMEDIS). Therefore, the data set is divided into 2 groups: *before* openMEDIS along 3 years and *after* openMEDIS along 3.25 years. Thus, openMEDIS acts as *a control barrier* by which risks can be mitigated.

In application, by utilizing the KPIs set for MIE management and by employing the data set for MIE, results are presented in Table 3. Considering all the proposed KPIs with their occurrence as shown in Table 1, each KPI is determined. For instance, KPI 1, *number of MIE with complaints*, is equal to 129 before, i.e., 43 per year, and 159 after, i.e., 49 per year. It is noticed that number of complaints is increasing after OpenMEDIS utilization, which is logical because equipment becomes older.

Comparing the proposed KPIs with data set, it is found that the main KPIs that play a significant role in risk control and summarize maintenance status are *six* KPIs. The KPIs are *number of CMVs*, *number of PMVs*, *total response time*, *total repair time*, *mean time between PMVs*, and *mean time between PMV and CMV*. The other ones are significant in performance evaluation.

**Figure 2:**
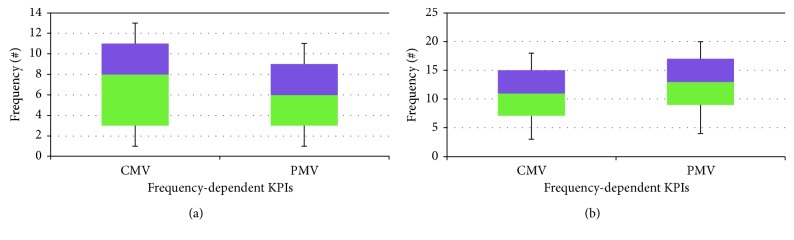
The box and whisker plot of two frequency-dependent KPIs: (a) before OpenMEDIS and (b) after OpenMEDIS.

**Figure 3:**
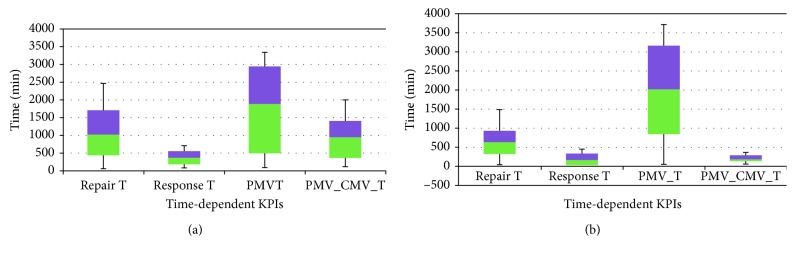
The box and whisker plot of four time-dependent KPIs: (a) before OpenMEDIS and (b) after OpenMEDIS.

A statistical analysis was carried out on main six KPIs including *median, minimum, maximum*, and *standard deviations* before and after OpenMEDIS as shown in Table 4. The box and whisker plot of these KPIs is depicted in Figures 2 and 3 as frequency-dependent and time-dependent, respectively. Each figure consists of 2 panels: *before* and *after* to illustrate the impact of the control barrier.  Figure 2 shows CMV and PMV, whereas the other KPIs are presented in Figure 3. Obviously, as shown in Figure 2, CMV and PMV are improved after OpenMEDIS. Like frequency-dependent KPIs, time-dependent KPIs are improved as presented in Figure 3 except *PMVT*.

**Figure 4:**
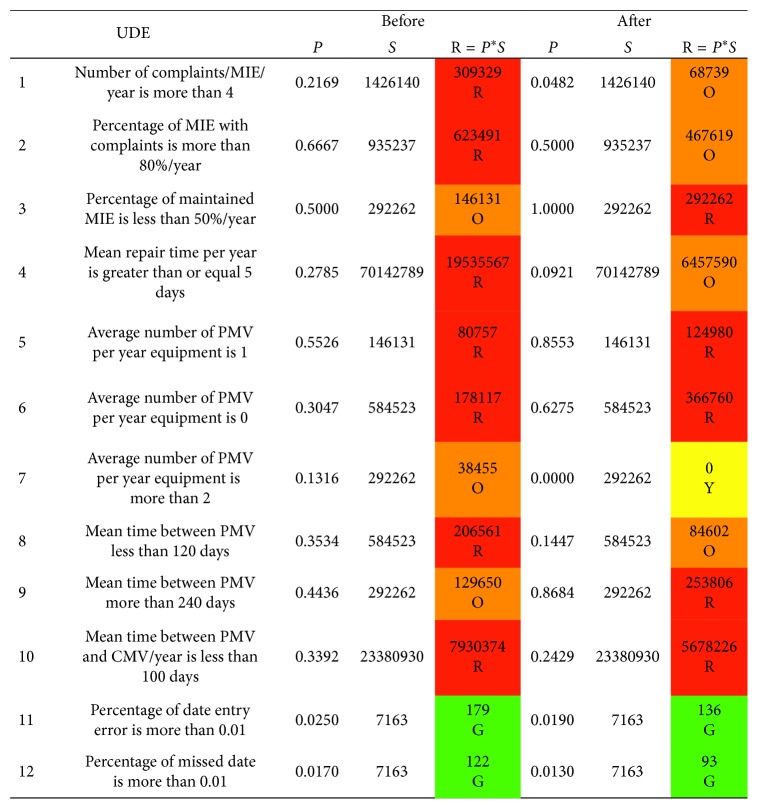
The risk matrix of UDEs before and after OpenMEDIS.

The risk level for each UDE is calculated and demonstrated in Figure 4. The risk number is presented *before* and *after* the control barrier. Visually as shown in Figure 4, some risk levels are transferred from unacceptable risk level (red) to manageable risk level (orange). The levels are related to *number of complaints per MIE per year*, *percentage of MIE with complaints*, *mean repair time per year*, and *mean time between PMVs is less than 120 days*. On contrast, few UDEs are transferred from manageable risk level to unacceptable level such as *percentage of maintained MIE* and *mean time between PMVs is more than 240 days*.

**Figure 5:**
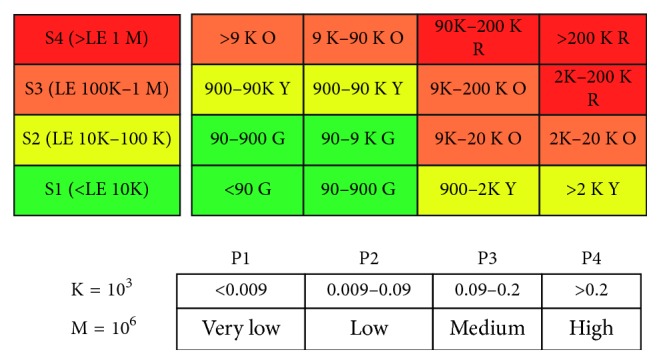
The risk matrix of the number of complaints for equipment per year, presenting the proposed scales of probability and severity.

Indeed, a risk matrix is established for every UDE. For instance, regarding *number of MIE with complaints per year*, there are 28 pieces that have the probability of occurrence of 0.2169 *before* using the control barrier, and *after* using it, the number of equipment becomes 8, which means the probability changed to be 0.0482. According to the proposed scale of probability of occurrence as shown in Figure 5, the probability of the event changed from *high* to *low*.

The severity is calculated as described in (1). By substituting with the *total cost* of all equipment 100,454,662 L.E. (75,529,145 L.E. for purchasing price, 20,304,553 L.E. for running costs, and 4,620,964 L.E. for hidden costs), the ADDC equals 4,848,876 L.E. Taking into account that the total number of equipment is 204 pieces, the MDDC is 23769 and MDC is 237690. Due to the history of MIE, the downtime was 6 days; hence, by using (1), the severity equals 1,426,140 L.E. Although the severity is in *a catastrophic* level *before* and *after* using the barrier, changing the probability leads to changing the risk level from unacceptable risk to conditionally acceptable risk as shown in Figure 5.

The same procedure is carried out for other UDEs to calculate the risk numbers and consequently the risk levels before and after the control barrier. As a result of this matrix, 36% of UDEs risk levels have been changed from unacceptable risk to conditionally acceptable risk, whereas 18% of UDEs risk level changed from conditionally acceptable to unacceptable that implies more analyses are required for significant control. Nevertheless, the risk level of others (46%) remains as the same before the barrier despite the probabilities of occurrence of some of them have been improved such as UDE10.

## 4. Conclusion

The study concerns risk analysis and risk control of medical imaging equipment management. Despite there is not a list of KPIs related to medical equipment management, a set of KPIs is proposed based on the maintenance standards to demonstrate risks associated with maintenance process. By using a control barrier, some risks are controlled to be mitigated, although others remain without changes, which reflect more features and deep analysis are required for risk control. For automation, the authors suggested cloud computing software as a control barrier.

This study highlights some criteria that pose risks if they are not well controlled such as *preventive maintenance frequency*, *registration of complaints*, and *mean repair time*. Moreover, measuring the severity of UDEs in a monetary value is more tangible and significant especially for developing countries due to their limited resources. Thus, optimizing risks with respect to costs seems influential in risk control.

Managing medical equipment based on a computerized management system is more appropriate because it facilitates equipment tracking by employing the registered data. Hence, risk management process becomes easier to be performed. Thus, full details of equipment history should be regarded in the inventory; otherwise, the proposed methodology will be limited during application. Moreover, a World Wide Web connection is an essential utility for implementation.

The proposed KPIs and UDEs could be expanded by adding more criteria to be implemented for other equipment in other departments and for other management processes such as acquisition. In addition, performance indicators are crucial elements in management process because it can measure the performance of individuals, equipment, and processes. The indicators act as proactive barriers that lead to risk reduction.

## Figures and Tables

**Table 1:** Proposed KPIs for MIE management.

Number	Class	KPI
1	E, C	Number of MIE with complaints/year
2	E, C	Number of complaints/MIE/year
3	E, C	Number of CMVs/MIE/year
4	C, P	Number of CMVs/complaint/year
5	P, C	Number of maintained MIE/year
6	P, C	Number of PMVs/maintained MIE/year
7	P, E, C	Mean response time/MIE/year
8	P, E, C	Mean repair time/MIE/year
9	P, E, C	Mean time between PMVs/MIE/year
10	P, E, C	Mean time between PMV and CMV/MIE/year
11	P, C	Percentage of detected data entry errors/year
12	P, C	Percentage of missed data/year

**Table 2:** Selected UDEs for MIE management.

Number	UDE	Threshold	Occurrence
1	Percentage of MIE with complaints/year	**>80%**	A/N
2	Number of complaints/MIE/year	**≥4**	UDE_2/K
3	Percentage of maintained MIE/year	**<50%**	M/N
4	Mean repair time/MIE/year	**≤5 days**	Days/T
5	Average number of PMVs/MIE/year	**≥2**	#PMV/M
6	Average number of PMVs/MIE/year	**≤1**	#PMV/M
7	Mean time between PMVs	**≤120 days**	B/M
8	Mean time between PMVs	**≥240 days**	C/M
9	Mean time between PMV and CMV/MIE/year	**≤100 days**	G/N
10	Percentage of detected data entry errors/year	**>1%** [13]	E/TD
11	Percentage of missed data/year	**>1%** [13]	MD/TD

**Table 3:** KPIs values before and after OpenMEDIS.

Number	KPI	Before	After
1	Number of MIE with complaints	129	159
	**Percentage of MIE with complaints/year**	**0.21**	**0.24**
2	Number of complaints/MIE	490	631
	**Number of complaints/MIE/year**	**1.27**	**1.22**
3	Number of CMVs/MIE	574	803
	**Number of CMVs/MIE/year**	**1.48**	**1.55**
4	Number of CMVs/complaint	1.17	1.27
	**Number of CMVs/complaint/year**	**0.39**	**0.39**
5	Number of maintained MIE	133	76
	**Percentage of maintained MIE/year**	**0.22**	**0.11**
6	Number of PMVs	246	87
	**Number of PMVs/maintained MIE/year**	**0.62**	**0.35**
7	Mean response time	19.65	6.15
	**Mean response time/year**	**6.55**	**1.89**
8	Mean repair time	240.06	79.81
	**Mean repair time/year**	**80.02**	**24.56**
9	Mean time between PMVs	667.29	939.08
	**Mean time between PMVs/year**	**222.43**	**288.95**
10	Mean time between PMV and CMV	712.17	271.28
	**Mean time between PMV and CMV/year**	**237.39**	**83.47**
11	Average number of detected data entry errors	0.077193	0.03269
	**Percentage of detected data entry errors/year**	**0.025731**	**0.010058**
12	Average number of missed data	0.529412	0.042857
	**Percentage of missed data/year**	**0.176471**	**0.013187**

**Table 4:** Statistical analysis summary of KPIs before and after OpenMEDIS.

KPI		Median	Min.	Max.	STD
CMV	Before	8	1	13	3.00
	After	11	3	18	2.74
PMV	Before	6	1	11	3.43
	After	13	4	20	2.92
Repair_T	Before	1320	350	2760	738.43
	After	657	60	1500	650.00
Response_T	Before	369	77	700	214.72
	After	215	88	498	85.17
PMV_T	Before	1900	100	3352	918.80
	After	2065	90	3760	906.01
PMV_CMV_T	Before	950	120	2000	540.56
	After	218	92	395	137.69
